# Biodegradable Magnesium Screws Accelerate Fibrous Tissue Mineralization at the Tendon-Bone Insertion in Anterior Cruciate Ligament Reconstruction Model of Rabbit

**DOI:** 10.1038/srep40369

**Published:** 2017-01-10

**Authors:** Jiali Wang, Jiankun Xu, Weimin Fu, Wenxiang Cheng, Kaiming Chan, Patrick Shu-hang Yung, Ling Qin

**Affiliations:** 1 Musculoskeletal Research Laboratory, Department of Orthopaedics & Traumatology, The Chinese University of Hong Kong, Hong Kong SAR, PR China; 2 Department of Orthopedics, Affiliated Zhongshan Hospital of Dalian University, Dalian, China; 3 Center for Translational Medicine Research and Development, Institute of Biomedical and Health Engineering, Chinese Academy of Sciences, Shenzhen 518055, PR China

## Abstract

The incorporation of tendon graft into bone tunnel is one of the most challenging clinical issues in anterior cruciate ligament (ACL) reconstruction. As a biodegradable metal, Mg has appropriate mechanical strength and osteoinductive effects, thus may be a promising alternative to commercialized products used for graft fixation. Therefore, it was hypothesized that Mg based interference screws would promote tendon graft-bone junction healing when compared to Ti screws. Herein, we compared the effects of Mg and Ti screws on tendon graft healing in rabbits with ACL reconstruction via histological, HR-pQCT and mechanical analysis. The histological results indicated that Mg screws significantly improved the graft healing quality via promoting mineralization at the tendon graft enthesis. Besides, Mg screws significantly promoted bone formation in the peri-screw region at the early healing stage. Importantly, Mg screws exhibited excellent corrosion resistance and the degradation of Mg screws did not induce bone tunnel widening. In tensile testing, there were no significant differences in the load to failure, stress, stiffness and absorption energy between Mg and Ti groups due to the failure mode at the midsubstance. Our findings demonstrate that Mg screws can promote tendon graft healing after ACL reconstruction, implying a potential alternative to Ti screws for clinical applications.

Injuries to the anterior cruciate ligament (ACL) are common for those individuals especially with high-level physical activity. Currently, 5 million new ACL ruptures are estimated to occur each year in the world^1^. More than half of the knees with ACL injuries are surgically reconstructed. Bone-patellar tendon-bone (BPTB) and soft tissue tendon graft, e.g. hamstring tendon, are the two most common alternative graft choices for ACL reconstruction^2^. According to current clinical reports, no significant differences were observed between BPTB and soft tissue grafts in clinic outcomes in terms of Lachman testing, chance of returning to the same level of sport, graft re-ruptures or other complications^3^,^4^. However, the use of BPTB graft may induce higher chance of donor-site morbidity^5^. Therefore, the use of soft tissue grafts has become increasingly popular in recent years^6^,^7^,^8^.

Currently, titanium (Ti) and degradable polymer based interference screws are the most commonly used tendon graft fixation devices in ACL reconstruction^9^,^10^. However, ACL reconstruction secures good to excellent results only in approximate 75% clinical cases^11^, which may be partially ascribed to poor integration of tendon graft into bone tunnel surface owning to the great differences between tendon graft and bone tissue in biochemical structure^1^,^12^. Increasing limitations have been generated in terms of the application of Ti and polymer devices in ACL reconstruction as a result of ultra-high rigidity of Ti metal and long-term acidic degradation products from polymers, which detrimentally affect graft healing^9^,^10^.

Injectable bone cements, growth factors and stem cells have been already reported as potential therapy options to promote tendon graft healing after surgery in animal models^13^. However, how to deliver them in the target region with optimized dose is still one of most challenging issues prior to implementation in clinic study. The use of biodegradable magnesium (Mg) based medical device, which has been intensively tested in preclinical and clinic studies as orthopaedic implants in the past decade owing to its appropriate mechanical strength and osteoinductive properties^14^,^15^,^16^,^17^,^18^,^19^,^20^,^21^,^22^, may be suitable for tendon graft fixation in ACL reconstruction as a new generation of bioactive implants. In aqueous solution, the degradation of Mg metal is initiated via its chemical reaction with water to release Mg ions and equivalent mole of hydrogen gas^23^. The promotive effects of Mg ions on osteogenic differentiation of stem cells may facilitate osseous ingrowth into tendon graft^24^, which can ultimately enhance the incorporation of tendon graft into surrounding bone tissue. Most recently, Cheng *et al*. reported that the use of Mg fixators may favor the formation of fibrocartilage at the intra-articular aperture in rabbits after ACL reconstruction^25^. Cheng *et al*. also found that Mg interference screws could effectively inhibit the degeneration of the tendon graft during the remodeling phase via reducing the expression level of MMP-13, indicating that more collagen fibers in the tendon graft were preserved to connect the surrounding bone tissue for higher knee stability^26^. However, it is still unclear if Mg based fixators can also promote graft healing at the mid-tunnel interface as spatial differences in tendon-bone healing in a bone tunnel was reported^27^. In addition, the lack of bone tunnel information after surgery is also a major clinical concern for the potential application of Mg fixators.

Therefore, it is of significance to investigate the effects of Mg implants on the tendon graft healing quality in the middle section and the alteration of bone tunnels in ACL reconstruction model. Herein, it was hypothesized that Mg based interference screw could significantly enhance the incorporation of tendon graft within a bone tunnel when compared to traditional Ti interference screws. To test the hypothesis specifically, ACL reconstruction surgery was performed in rabbits by using Mg or Ti based interference screws for histological, radiographic and mechanical testing at different time points.

## Results

### The use of Mg interference screws does not alter serum Mg levels in rabbits

**Figure 1:**
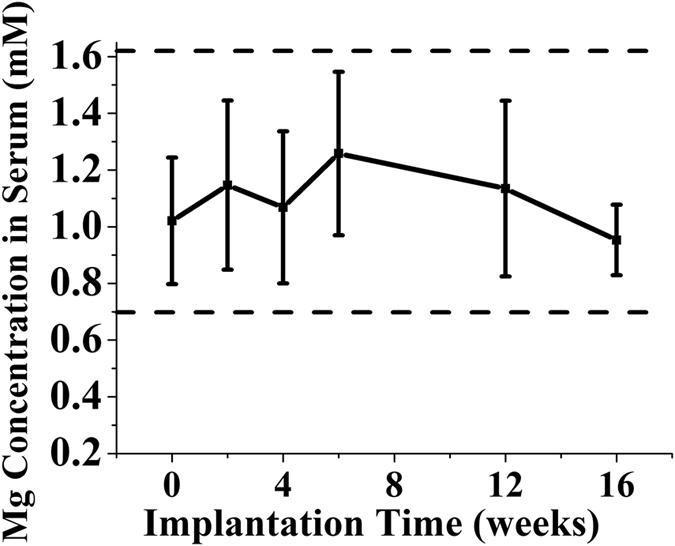
Serum Mg levels in rabbits at all examined time points during the 12 weeks after ACL reconstruction by using Mg interference screws and the reference range for serum Mg ion concentration in rabbits.

The serum Mg levels in testing animals were found within the normal reference range at all examined time points during the four month implantation period^28^. In addition, the absence of temporal changes in serum Mg levels was detected (Fig. 1).

### The degradation of Mg interference screws does not increase serum levels of IL-1 and IL-6

**Figure 2:**
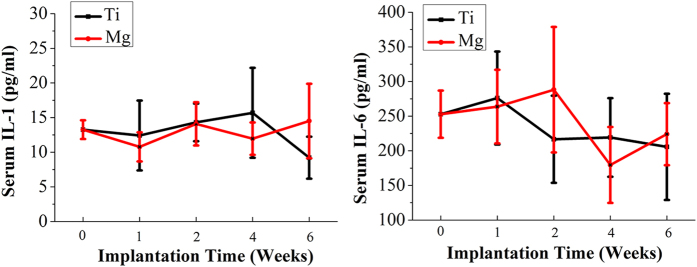
Serum IL-1 and IL-6 levels in rabbits at week 0 (baseline), 1, 2, 4 and 6 after surgery.

As shown in Fig. 2, there were no significant differences in serum levels of the IL-1 and IL-6 between Mg group and Ti group at week 0, 1, 2, 4 and 6 postsurgically. Additionally, there were no significant temporal changes of IL-1 and IL-6 levels in Mg or Ti groups.

### Mg interference screws promote tendon graft osseointegration via enhancement of fibrous tissue mineralization at graft enthesis

**Figure 3:**
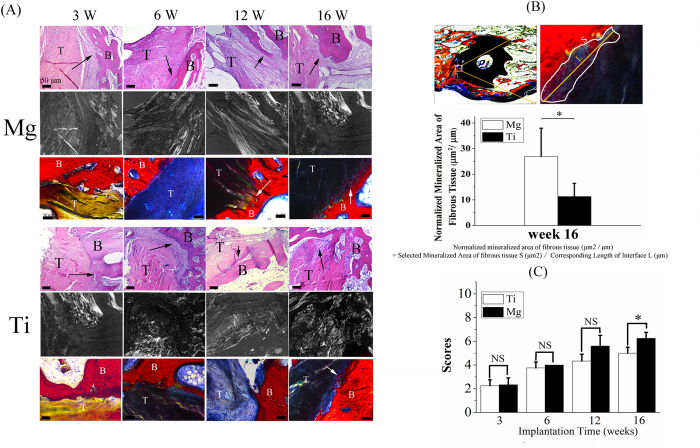
Histological examination of tendon-bone junction healing quality in Mg and Ti groups over time. (**A**) H&E and Stevenel blue-Van Gieson-Alizarin Red S stained femoral tunnel under bright field and polarized illumination for evaluation of tendon graft-bone junction healing at week 3, 6, 12 and 16 after reconstruction. The fibrous tissue in the interzone was indicated by the black arrow in H&E stained samples while the mineralized fibrous tissue was labeled by the white arrow in Stevenel blue-Van Gieson-Alizarin Red S stained samples. T: tendon graft; B: bone. Scale bar is 50 μm. (**B**) Representative images on how to calculate normalized mineralized area (top) and calculated values assigned for Mg and Ti groups at week 16 postsurgically. The mineralized area in the interface between the tendon graft and bone increased significantly in Mg group compared to Ti group. S: area of mineralized fibrous tissue; L: the length of corresponding mineralized interface. n = 4, *P < 0.05 (non-parametric Mann-Whitney U test). (**C**) The sum of histological score for evaluation of tendon graft-bone junction healing quality. n = 4, *P < 0.05, NS: *P* > 0.05 (non-parametric Mann-Whitney U test).

The fibrous tissue with massive cellularity and vascularity was formed and uniformly distributed in the mid-tunnel interface between tendon graft and bone as early as 3 weeks after surgery and then increased over time within the first 12 weeks in both Ti and Mg groups (Fig. 3A). At 16 weeks postoperatively, the area of fibrous tissue interface decreased while the collagen fibers in the fibrous tissue became more aligned, which indicated the remodeling phase of graft healing. The mineralization of fibrous entheses was detected at week 12 in Mg group while at week 16 in Ti group (Fig. 3A). More importantly, the area of mineralized fibrous tissue was significantly increased in Mg group when compared to Ti group (27.1 ± 10.4 μm^2^/μm vs. 12.1 ± 3.9 μm^2^/μm) at week 16 (Fig. 3B). The results from a semi-quantitative histological scoring system showed that Mg group had a significant improvement in graft healing quality at week 16 compared with Ti group (Fig. 3C).

### Mg interference screws promote new bone formation in the peri-screw region at early time point

**Figure 4:**
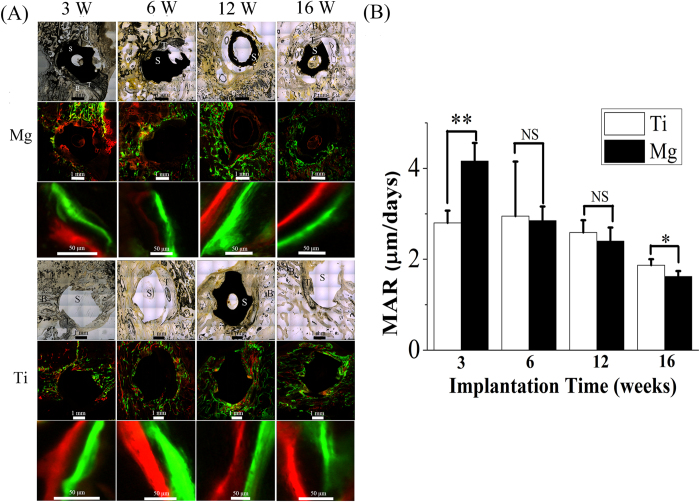
The representative images showing the mineral apposition rate (MAR) in surrounding bone tissue in femur around Ti or Mg screws. (**A**) White light photographic and fluorescent images of tendon graft-screw-bone complex at different time points for both Mg and Ti groups. T, S and B represent tendon graft, screw and bone, respectively. (**B**) The measured MAR values in Mg and Ti groups assigned for different time points. n = 4, *P < 0.05, **P < 0.01, NS: *P* > 0.05 (non-parametric Mann-Whitney U test).

The mineral apposition rate (MAR) in bone tissue around screws in Mg group was significantly higher than that in Ti group at week 3 (4.2 ± 0.4 μm/day vs. 2.80 ± 0.27 μm/day, *P* < 0.01), indicating an acceleration of bone remodeling after the use of Mg screws. In the following healing stages, MAR values were then reduced in both Mg and Ti groups (Fig. 4).

### The degradation of Mg interference screw does not cause enlargement in bone tunnel

**Figure 5:**
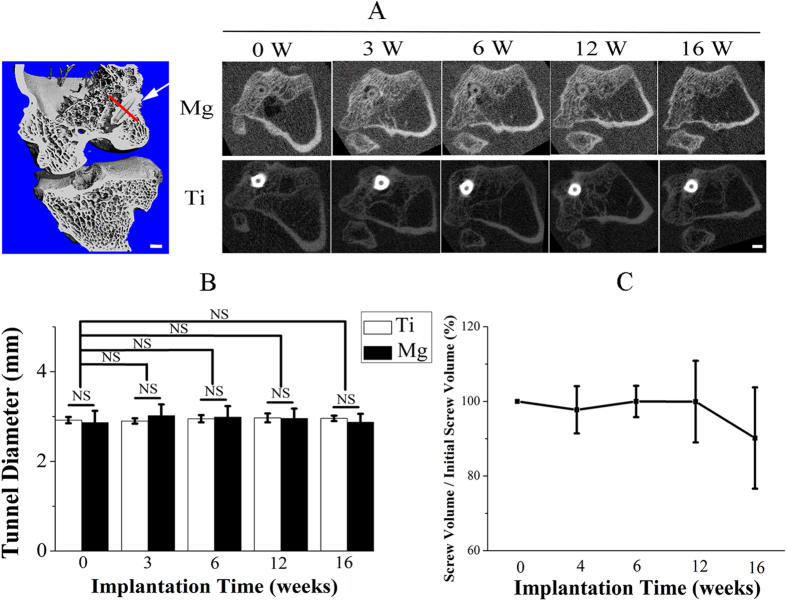
Representative CT scanning of the femur-tendon graft-tibia complex (FTGTC) in rabbits with the insertion of Mg or Ti interference screw. (**A**) The longitudinal section image (left: 3D image) of FTGTC and the transverse section image at the red line perpendicular to interference screws (indicated by white arrow) in the 3D model reconstructed from a series of HR-pQCT scan images of FTGTC. Scale bar is 2 mm. (**B**) Temporal changes in the diameter of femoral tunnels in both Mg and Ti groups. n = 6, NS: *P* > 0.05 (one-way ANOVA with Tukey’s *post hoc* test). (**C**) Temporal changes of the percentage in the remaining volume of Mg screws. n = 6.

The use of degradable Mg and Ti screws did not induce obvious bone tunnel widening even in the early healing stage (Fig. 5A). The tunnel diameter at week 3 and 6 was relatively higher (4.9% and 4.2% increase, respectively) than baseline in Mg group (*P* > 0.05) while the gap between Mg screws and bone was gradually refilled by newly formed bone tissue at week 12 and 16 (Fig. 5B). Tunnel size remained unchanged postoperatively in Ti group. The degradation of Mg interference screws was not accompanied with gas accumulation in peri-implant cavity. Approximate 10% loss in the apparent volume of Mg screws was detected within the entire implantation period, but there were no significant differences in the volume of Mg screws within any time points, suggesting excellent corrosion resistance for our used high-purity Mg screws (Fig. 5C).

### Both Mg and Ti interference screws induce bone loss in peri-tunnel bone tissue

**Figure 6:**
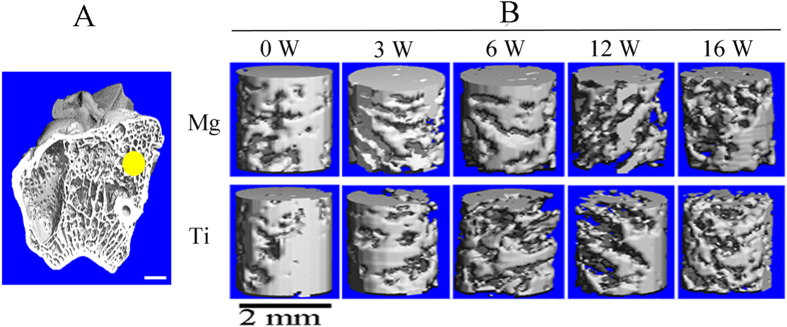
Representative CT scanning of rabbit femur for the measurement of peri-screw trabecular bone. (**A**) The outlined peri-tunnel bone tissue by yellow circle in femoral side for 3D reconstruction of 80 slices in the middle section at week 0, 3, 6, 12 and 16 post-surgically; (**B**) The 3D model of reconstructed trabecular bone tissue for comparison of related parameters, i.e. BV/TV and bone mineral density (BMD) in Mg and Ti groups at week 0, 3, 6, 12 and 16 after surgery. Scale bar is 2 mm.

As shown in Fig. 6, the ratio of bone volume/tissue volume (BV/TV) in peri-tunnel bone tissue was significantly reduced in both Mg and Ti groups within the initial 12 weeks after reconstruction (Table 1). The bone mineral density (BMD) of peri-tunnel bone did not change significantly in both Mg and Ti groups during the entire experimental period (Table 1).

### Mg interference screws provide sufficient fixation ability to support tendon graft healing

**Figure 7:**
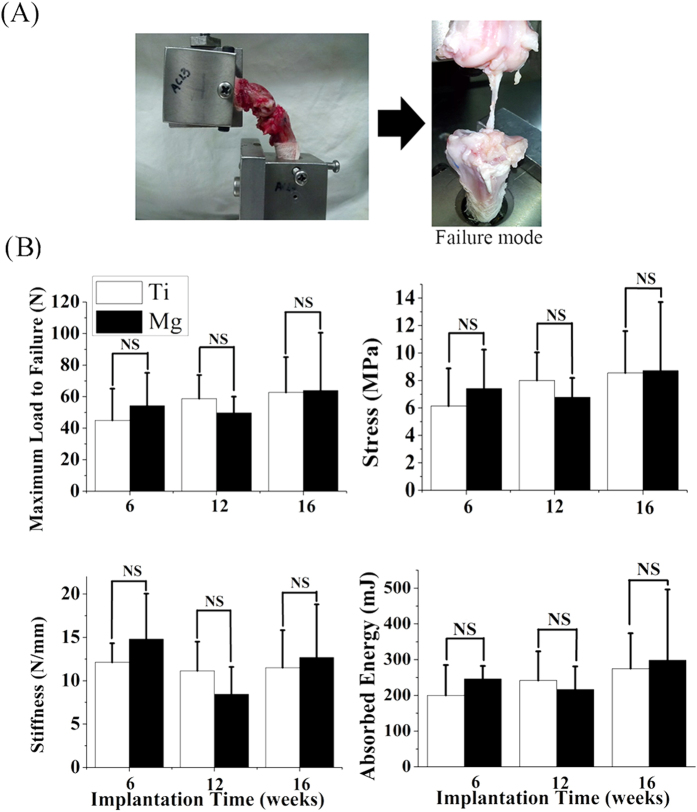
Tensile test of FTGTC using a custom-designed jig conducted at 6, 12 and 16 weeks after ACL reconstruction surgery in rabbits. (**A**) All the failure occurred at graft midsubstance (right) for tested FTGTC samples fixed in the testing jig at a knee flexion angel of 90. (**B**) The maximal failure load, stiffness, stress and absorption energy in FTGTC samples in tensile testing in Mg and Ti groups. n = 8 for each group, NS: *P* > 0.05 (unpaired Student’s *t*-test).

At week 6, 12 and 16 postsurgically, the failure occurred at midsubstance in both Mg and Ti groups during tensile loading tests (Fig. 7A). There were no significant differences in load to failure (70.7 ± 34.1 N in Mg group vs. 62.7 ± 22.3 N in Ti group), stiffness (13.3 ± 6.3 N/mm in Mg group vs. 11.5 ± 4.3 N/mm in Ti group), stress (9.6 ± 4.9 in Mg group vs. 8.5 ± 3.0 MPa in Ti group) and energy (298.3 ± 198.0 mJ in Mg group vs. 275.0 ± 98.6 mJ in Ti group) between Mg and Ti groups at week 16 (Fig. 7B). Similarly, Mg group did not show significant differences in the values of load to failure, stiffness, stress and energy compared with Ti group at week 6 and 12 (Fig. 7B).

## Discussion

Poor tendon graft incorporation into the bone tunnel has been widely considered as one of the main causes leading to nontraumatic ACLR failure^29^,^30^. Our present *in vivo* study results clearly demonstrated that the use of Mg based interference screws can effectively enhance tendon-bone junction healing via promoting tendon graft enthesis mineralization in ACL reconstruction model without biosafety concerns.

Actually, Cheng *et al*. has also recently used Mg interference screws to fix tendon graft into bone tunnels in a rabbit model of ACL reconstruction^26^. Although our animal model is similar to Cheng’s model, the research interests of Cheng PF’s and our studies are different. Briefly, Cheng mainly investigated the role of Mg implants in inhibiting tendon graft degradation while our work focused on the effects of Mg implants on graft-bone interface healing. In Cheng’s work, they found that Mg interference screws could downregulate the expression of MMP-13 and preserve collagen fibers at the tendon graft, which may ultimately contribute to the enhancement of tendon graft healing. In our work, we studied the biological effects of Mg implants on tendon-bone junction healing instead of graft changes. Our histological data validated that Mg implants can accelerate and significantly promote the mineralization of the interzone structure between tendon graft and bone tissue, indicating that Mg interference screws can enhance graft integration into bone tunnels after ACL reconstruction. Although both of our and Cheng’s work validated the superior advantages of Mg interference screws to traditional fixators in ACL reconstruction, we elucidated the mechanisms from different directions. According to Cheng’s findings, the inhibition of graft degradation by Mg screws was beneficial to the regeneration of highly differentiated Sharpey-like fibers in direct apposition with bone, which may improve graft bonding strength and its stability during remodeling stage. Our data showed that more calcified fibrous tissue could be formed in the interface under the stimuli of Mg screws, indicating optimized interzone structure (fibrous tissue and calcified fibrous tissue in the interface) to bridge to gap between tendon graft and bone tunnel surface for load transfer with reduced re-injury concerns^31^,^32^. In addition, we have studied the effects of Mg implants on bone tunnels as the enlargement of tunnels is also a clinical concern in ACL reconstruction.

More importantly, as spatial differences in the graft healing was reported^27^, so the tendon-bone attachment site in the mid-tunnel instead of the intra-articular aperture (Cheng’s work) was selected for evaluation of the graft healing quality in our study. The tendon graft healing was enhanced within the entire experimental period according to the histological semi-quantitative scoring evaluation, which was similar to Kuang’s reported findings^33^. In the late healing stage, i.e. 16 weeks after surgery, a significantly larger area of calcified fibrous tissue was formed in the interface of Mg group when compared to Ti group, which can improve the incorporation strength of the healing graft into the surrounding bone after the use of Mg screws.

The repair effects of Mg implants on the tendon graft healing in ACL reconstruction may be ascribed to 3 possible causes. Firstly, the released Mg ions from implants have been reported to activate both osteoclast and osteoblast function for promoted bone remodeling, so the increased bone resorption in peri-implant may positively stimulate the release of transforming growth factor-β1 (TGF-β1)^34^, which can induce the migration of bone marrow stem cells (BMSCs) for neo-tissue growth. Concomitantly, the stimuli of Mg ions may up-regulate the number of precursors of osteoclasts^35^, so more platelet derived growth factor (PDGF-BB) from preosteoclasts can be secreted to mobilize BMSCs towards the resorption sites^36^. Therefore, the use of Mg based interference screws may recruit more BMSCs in the interface. In addition, the released Mg ions from implants was reported to significantly increase cell adhesion owning to the promoted α5β1- and β1-integrin expression levels^37^,^38^. As the osteogenic differentiation capability of BMSCs was significantly enhanced in higher Mg ion levels (Supplementary Fig. S1), the migrated BMSCs towards the tendon graft entheses with enhanced adhesion ability on the graft surface may also facilitate osseous ingrowth into the graft insertion site, contributing to higher bonding strength between the tendon graft and bone.

Although Mg based screws showed favorable biological effects via *in situ* release of degradation products from implants, the deterioration manner in the mechanical integrity of screws may well match the tendon graft healing process without fixation concerns. Only 10% loss in the apparent volume of Mg screws was observed after 16 weeks. More importantly, the degradation of Mg screws did not induce bone tunnel widening during the entire experimental period. As the low production of artifact caused by the Ti hardware on imaging did not significantly alter the bone related parameters (Supplementary Table S1), including BV/TV (Ti implantation vs. Ti removal: 0.354 ± 0.026 vs. 0.337 ± 0.062, *P* = 0.496) and BMD (1.882 ± 0.027 vs. 1.881 ± 0.009, *P* = 0.842), CT analysis was suitable for the assessment of temporal change of peri-tunnel bone quality in both Mg and Ti groups. The peri-tunnel bone mass, which is commonly reduced after ACL reconstruction in both preclinical and clinical cases^39^, was also observed deterioration in both Mg and Ti groups after surgery, which may be ascribed to the promoted expression of MMP1, MMP13 and CD68+ cells at the peri-tunnel region during the graft healing stage^40^. The peri-tunnel bone loss mainly occurred within the initial 12 weeks after surgery, indicating that 12 weeks postsurgically is the critical time point affecting knee function restoration.

However, the direct mechanical proof on graft bonding strength was not delineated as the failure sites of all the femur-tendon graft-tibia complex (FTGTC) samples in tensile testing were in the tendon graft midsubstance in both Mg and Ti groups. Actually, the primary mode of failure was widely reported at midsubstance after reconstruction in animals especially in the long-term study^39^,^41^,^42^,^43^. Our tested mechanical results at week 6, 12 and 16 after surgery indicated that sufficient graft integration can be formed by using conventional Ti-based or innovative biodegradable Mg-based interference screws in rabbits for fixation of the tendon graft in ACL reconstruction model.

However, there are several limitations in the present study. Firstly, as the metallic screws may easily cause damage to the soft-tissue grafts owing to their sharp edges, biodegradable polymer screws may be theoretically more favorable for fixation of soft-tissue grafts^44^. In this respect, polymer interference screws may be more comparable to Mg screws in this study. Besides, although we observed that the degradation of Mg screws did not trigger any adverse effects on the graft healing within 16 weeks postsurgically, it is still unclear if the degradation products from Mg screws would impair tendon graft-bone junction structure in the longer term. It is necessary for us to extend the observation time in the future study. Lastly, the limitation of smallest voxel size in HR-pQCT may affect trabecular bone results. Generally, the voxel size for a μCT scan can strongly affect trabecular or cortical bone results if the voxel size is not appropriately small compared to the dimensions of the structure being measured^45^. Scanning with low resolution (large voxel size) relative to the size of the trabecular bone can cause an underestimation of bone mineral density due to partial-volume effects and overestimation of BV/TV, trabecular thickness (Tb.Th) and trabecular separation (Tb.Sp)^45^,^46^,^47^. The trabecular thickness in adult rabbits is approximate 180 μm^48^, while the smallest voxel size is 41 μm in our used HR-pQCT. Therefore, the voxel size to trabecular thickness in our *ex vivo* CT scans of rabbits is about 1:4.5. Christiansen’s findings showed that the ratio of voxel size to trabecular thickness between 1:4 to 1:6 did not significantly affect BV/TV^47^. However, tissue BMD was significantly reduced from 880 mg HA/cm^3^ to approximate 820 mg HA/cm^3^ when the ratio of voxel size to trabecular thickness increased from 1:6 to 1:4. Therefore, BMD values may be underestimated while BV/TV may not be affected in trabecular outcomes for scans with 41 μm voxel size in our study. Although the relatively large voxel size is the limitation of HR-pQCT when applied to scan smaller structures, the *ex vivo* scanning model can effectively address the concerns about the inter-group variation. Currently, the smallest voxel size in the updated HR-pQCT devices can be down to 17 μm, indicating that we can get more satisfactory results in the future study if the conditions are met.

In summary, the present study suggested that the Mg based biodegradable interference screws might be an alternative to traditional inert fixators for treating tendon graft-bone junction healing in ACL reconstruction owing to the promoted mineralization at the fibrous entheses. In addition, the degradation products of Mg implants did not cause significant changes in bone tunnel size and inflammatory responses in rabbits. The biosafety and bio-efficacy experiments have been tested and guaranteed in small animals, so it is of significance to extend the research in large animals for the ultimate translation of Mg screws in clinical trials.

## Materials and Methods

### Animal Surgery

**Figure 8:**
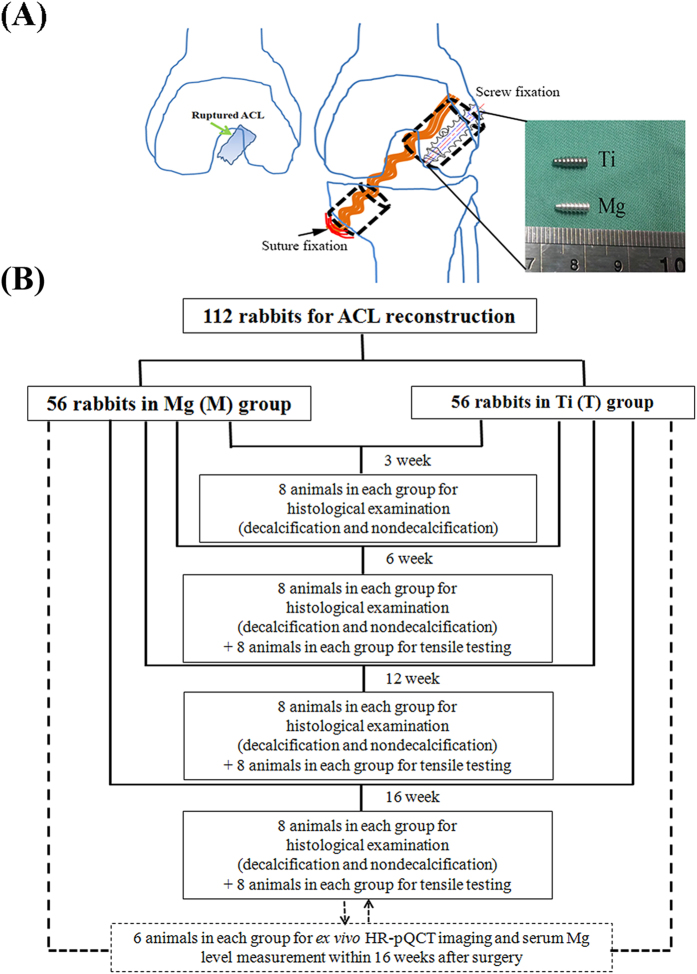
Surgery description and experimental design. (**A**) The schematic diagram showing the surgical procedures of ACL reconstruction in rabbits by using Mg or Ti interference screws of same size and design for comparison; (**B**) Flow chart for experimental design demonstrating animal grouping and testing at each time point. HR-pQCT: High resolution-peripheral quantitative computed tomography.

A total of 112 ACL reconstruction procedures were performed in left knees of 6-month-old male New Zealand White rabbits by using the long digital extensor tendon autograft via transtibial technique according to our established experimental protocol approved by the Animal Ethics Committee of the Chinese University of Hong Kong (13/041-MIS-5), which was in accordance with the guidelines for the ethical treatment of animals (Fig. 8A). The surgical protocols were approved by the animal experimentation ethics committee (AEEC). Briefly, the bone tunnels with the diameter of 2.5 mm in both the femur and tibia were created along the direction of the ACL footprint by a hand drill to allow the placement of the graft for the replacement of the transected ACL. Then the high purity Mg (99.99 wt.%) or commercial Ti interference screws with 3.0 mm in diameter and 8.0 mm in length were inserted into the femoral tunnels from extra-articular exit to fix the graft, which may minimize the effects of synovial fluid on degradation of Mg implants. Comparably, the non-absorbable suture instead of screws was used to tightly suture the other end of the graft in tibial tunnel’s exit in surrounding soft tissue due to insufficient trabecular bone in proximal tibia. The tension was applied to the graft during the entire period for graft fixation. The animals after reconstruction were kept in cage for free movement without knee immobilization. At week 3, 8 animals from each group were terminated by administration of overdosed pentobarbital sodium to harvest femur-tendon graft-tibia complex (FTGTC) samples for histological analysis (n = 8 per each group per time point). At week 6, 12 and 16 after surgery, the remaining 96 animals were sacrificed for both histological examination (n = 8 per each group per time point) and mechanical testing (n = 8 per each group per time point). The flow chart for the experimental design was shown in Fig. 8B.

### Serum Mg levels after reconstruction

The blood samples of rabbits in Mg group were collected at week 0 (baseline), 2, 4, 6, 12 and 16 postsurgically for serum Mg level determination by using inductively coupled plasma-mass spectroscopy (ICP-MS, Agilent Technologies, Tokyo, Japan).

### Serum interleukin-1 (IL-1) and IL-6 levels after reconstruction

IL-1 and IL-6 levels were measured in serum of the rabbits after the use Mg and Ti screws at week 0 (baseline), 1, 2, 4 and 6 by means of ELISA (Enzyme Linked-Immuno-Sorbent Assay) method by using commercial kits (Neoscientific, MA, USA).

### Fluorochrome sequential labeling of mineralized tissue around the implants

To label newly formed bone, the fluorescent staining agent calcein green (10 mg/kg) was subcutaneously injected into these animals assigned for four time points at 14, 28, 63 and 84 days postsurgically prior to xylenol orange (90 mg/kg) injection at 21, 42, 84 and 112 days after surgery, respectively. All the rabbits were then sacrificed for sample harvest at day 3 after xylenol orange injection. Mineral apposition rate (MAR) in the bone tissue for the region of interest (ROI) adjacent to the implants was calculated to compare the apposition rates at different healing stages according to the standardized definition^49^.

### Histological analysis

Both decalcified and undecalcified samples were used for histological examination under a microscope (Leica DM 5500B, Germany). Briefly, all these harvested femora samples were fixed in 4% neutral buffer formalin for 48 h and then embedded in paraffin or methylmethacrylate (MMA) according to previously published protocols prior to tissue sectioning at mid-tunnel site^41^. As the general inspection method for histological analysis, hematoxylin & eosin (H&E) staining was performed for all the sections to evaluate the healing taking place between tendon graft and bone at the defined time points^50^. Tendon graft-bone junction healing quality was assessed according to our modified histological scoring system originally proposed by Lui *et al*.^51^ with 5 items of histologic features, including interface region area ratio in graft, graft remodeling status, graft collagen orientation, tendon-bone connection direction and interzone mineralization (Table 2). Stevenel blue-Van Gieson-Alizarin Red S staining was performed on the thick MMA sections with approximate 100 μm according to the commonly used protocols^52^. Briefly, MMA embedded sections were immersed into the 60 °C Stevenel’s blue solution for 15 min prior to Van Gieson’s stain for 5 min at room temperature. Then 2% Alirazin Red S solution was used to stain samples for another 5 min. Finally, MMA sections were washed thoroughly in running water to remove excess stain and kept dry in the air for microscopic imaging at the graft enthesis for quantitative analysis of mineralized area. In addition, the MMA cross-sections were observed by light microscopy and fluorescence microscopy, respectively.

### Radiographic imaging for femoral tunnels and the apparent volume of Mg screws via high-resolution peripheral quantitative computer tomography (HR-pQCT)

**Figure 9:**
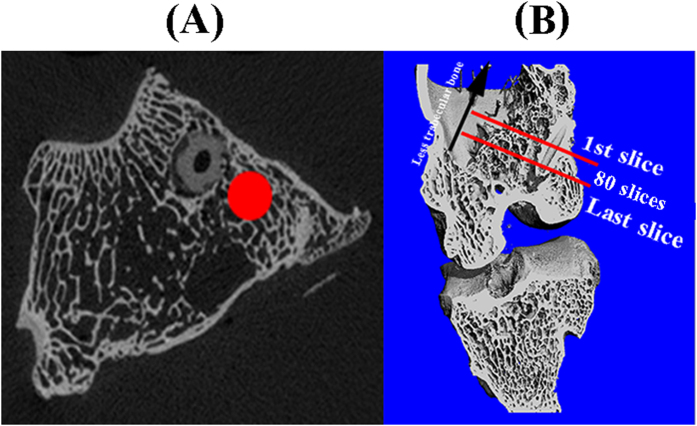
(**A**) The selection of ROI (labeled by red circle) in gray-images for 3D reconstruction of peri-tunnel trabecular bone. (**B**) A total number of 80 slices were segmented for creating 3D models of ROIs outlined in (**A**).

Six rabbits were anesthetized and immobilized in the scanner tube of HR-pQCT (Scanco Medical AG, Brüttisellen, Switzerland) for scanning their knees at week 0, 3, 6, 12 and 16 after operation. The fixed parameters for X ray-tube were set: 60 kV (tube voltage), 1 mA (tube current), 41 μm (an isotropic voxel size). A series of 2D tomographic gray-images (80 slices) in the mid-tunnel along the direction perpendicular to the screws were segmented for 3D reconstruction of peri-tunnel trabecular bone (Fig. 9). In order to standardize CT analysis protocol, the 2D gray-image with the cross-section of screws close to the extra-articular exit of femoral tunnels is defined as the first slice (Fig. 9A). As the trabecular bone is reduced gradually along the direction from intra-articular site to extra-articular site (Fig. 9B), it is not recommended to select a larger region of interest (ROI). Otherwise, cavity instead of trabecular bone will be included into ROIs for 3D reconstruction, which may ultimately dilute the potential difference between Ti group and Mg group with regard to the effects of used screws on the surrounding bone tissue. Therefore, we only selected 2 mm region for 3D reconstruction in this work (Fig. 9B). In addition, the degradation rate of Mg screws over time was also calculated based on 3D reconstructed models. Prior to 3D reconstruction of selected Volume of Interest (VOI) in both implants and bone tissue, the resulting gray-images were segmented using a fixed threshold and a low-pass filter to minimize the noise (Sigma = 1.2, support = 2.0, threshold = 145). Therefore, bone tunnel diameter (mm), the relative degradation rate of Mg screw, BV/TV and BMD (mg HA/ccm) of peri-tunnel bone tissue were calculated.

### Biomechanical testing

The femur-tendon graft-tibia complexes (FTGTC) were harvested and then stored in −80 °C prior to biomechanical testing after thawing at room temperature. Except for the ACL graft, the suture on the tibial side and all the soft tissue were carefully removed while interference screws were kept in femoral tunnels prior to the fixation of femur and tibia with custom-designed jigs in a uniaxial mechanical testing machine (H25K-S, Hounsfield Test Equipment LTD, Surrey, UK). The tensile tests were performed in the femur-ACL graft-tibia complex with the knee flexed to 90° according to previously published protocols with a preload of 1 N and a rate of 50 mm/min until failure^41^. Before the tensile test, the cross-sectional area (CSA) of graft midsubstance was measured by a high-resolution ultrasound imaging system (Vevo 770, VisualSonics, Canada). Precision error was determined by measuring CSA values 6 times repeatedly for calculating coefficient of variation (CV)^15^ that showed 5.9%. The peak load, stiffness, stress and site of failure or failure mode were recorded for all samples. Eight samples were used for each group.

### Statistical Analysis

The non-parametric Mann-Whitney U-test was performed for calculation of significant differences in mineralized area in graft healing interface, histological assessment scores and MAR between Mg and Ti groups while one-way ANOVA with Tukey’s *post hoc* test was applied for statistical analysis of BV/TV, BMD, serum IL-1 and IL-6 levels in the two groups by using SPSS 17.0 software (SPSS Inc. Chicago, IL). The unpaired two-tailed Student’s *t*-test was used to compare the mechanical data between Mg and Ti groups. All the data were expressed as mean ± standard deviation (SD) with the significant level set as *P* < 0.05.

## Additional Information

**How to cite this article**: Wang, J. *et al*. Biodegradable Magnesium Screws Accelerate Fibrous Tissue Mineralization at the Tendon-Bone Insertion in Anterior Cruciate Ligament Reconstruction Model of Rabbit. *Sci. Rep.* **7**, 40369; doi: 10.1038/srep40369 (2017).

**Publisher's note:** Springer Nature remains neutral with regard to jurisdictional claims in published maps and institutional affiliations.

## Supplementary Material

Supplementary Information

## Figures and Tables

**Table 1:** HR-pQCT derived peri-tunnel trabecular bone parameters (Mean ± SD, n = 6).

Group	ROI	Bone parameters	Time point after ACL surgical reconstruction (weeks)				
			0	3	6	12	16
Mg	Peri-tunnel Bone Tissue	BV/TV	0.59 ± 0.13	0.46 ± 0.077	0.51 ± 0.095	0.39 ± 0.10*	0.39 ± 0.09
		BMD (mg HA/ccm)	589.66 ± 38.16	559.66 ± 16.77	564.66 ± 26.07	559.50 ± 20.32	567.50 ± 20.32
Ti		BV/TV	0.60 ± 0.13	0.52 ± 0.12	0.51 ± 0.09	0.32 ± 0.11*	0.39 ± 0.11
		BMD (mg HA/ccm)	588.05 ± 15.53	586.66 ± 28.55	593.83 ± 22.60	567.83 ± 28.68	597.00 ± 15.32

Note: *P < 0.05 compared to values at time zero, analyzed using one-way ANOVA followed up with Tukey’s post-hoc test (n = 6). ROI: Region of interests of HR-pQCT scanning; Mg: Magnesium; Ti: Titanium; BMD: Bone mineral density; BV: Bone volume; TV: Tissue volume.

**Table 2:** Histological scoring system for evaluation of tendon-bone junction healing quality.

Histological features	Score
**Area ratio of interface in graft**	
Massive (>50% in the graft area)	2
Present (<50% in the graft area)	1
None (0% in the graft area)	0
**Collagen orientation**	
Massive (>50% in the interface)	2
Present (<50% in the interface)	1
None (0% in the interface)	0
**Head to Head connection**	
Massive (>50% in the interface)	2
Present (<50% in the interface)	1
None (0% in the interface)	0
**Graft remodeling**	
Massive (>50% of graft remnant)	2
Present (<50% of graft remnant)	1
None (0% of graft remnant)	0
Interzone mineralization*	
Massive (>20 μm^2^/μm)	2
Present (<20 μm^2^/μm)	1
None	0
**Maximal sum of score**	10

*The mineralized area in the fibrous tissue bridging the bone and the tendon graft in Mg group was significantly higher than that in Ti group at week 16 after surgery (27.1 ± 10.4 μm^2^/μm vs. 12.1 ± 3.9 μm^2^/μm), so 20 μm^2^/μm was considered as the reference value.
