# Supplementary material for: Biodegradable Magnesium Screws Accelerate Fibrous Tissue Mineralization at the Tendon-Bone Insertion in Anterior Cruciate Ligament Reconstruction Model of Rabbit
Source: Sci Rep. 2017 Jan 10;7:40369. doi: 10.1038/srep40369 (PMC5223185; doi:10.1038/srep40369)
Supplement: Supplementary Information [file srep40369-s1.pdf]

Biodegradable Magnesium Screws Accelerate Fibrous Tissue Mineralization at the Tendon-Bone Insertion in Anterior Cruciate Ligament Reconstruction Model of Rabbit

Jiali Wang [a, #], Jiankun Xu [a, #], Weimin Fu [b], Wenxiang Cheng [c], Kaiming Chan [a], Patrick Shu-hang Yung [a], Ling Qin [a, c, *]

[a] Musculoskeletal Research Laboratory, Department of Orthopaedics & Traumatology, The Chinese University of Hong Kong, Hong Kong SAR, PR China

[b] Department of Orthopedics, Affiliated Zhongshan Hospital of Dalian University, Dalian, China

[c] Center for Translational Medicine Research and Development, Institute of Biomedical and Health Engineering, Chinese Academy of Sciences, Shenzhen 518055, PR China

Supplementary Figures

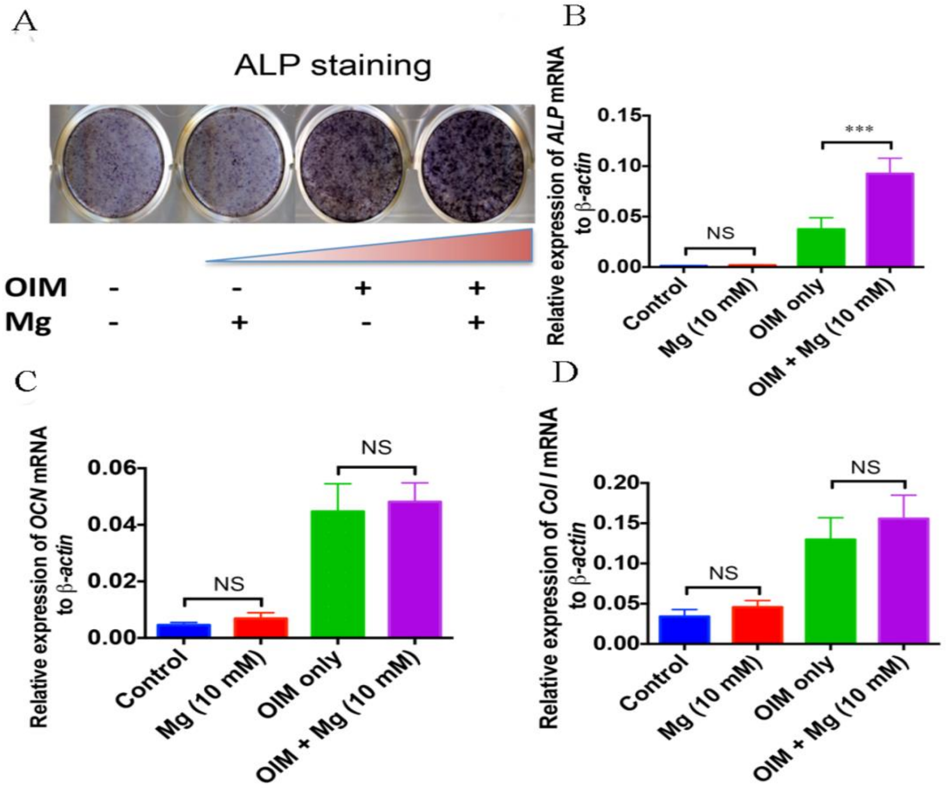

Figure S1 Osteogenic differentiation of BMSC cultured in basal complete medium and osteogenic induction medium (OIM) containing normal or 10 mM Mg ion level. (A) ALP staining of cultured BMSC at day 14. Higher Mg dose in OIM enhanced ALP activity of BMSC. (B-D) mRNA expression levels of three osteogenic

---

*Corresponding author. Musculoskeletal Research Laboratory, Department of Orthopaedics & Traumatology, Room 74034, Prince of Wales Hospital, The Chinese University of Hong Kong, Hong Kong SAR, PR China. Tel: +852-26323071; fax: +852-26324618.

E-mail address: qin@ort.cuhk.edu.hk.

#The authors contributed equally to this work.

differentiation related genes (*ALP, OCN* and *Col I*) in BMSC at day 14. Enhanced expression of *ALP* mRNA was observed in BMSC cultured in OIM added with 10 mM Mg ions, as compared to OIM only group. n=3, ****P*<0.001, NS: not significant (unpaired Student's *t*-test).

Supplementary Tables

Table S1 The effects of Ti screws on bone parameters

	Samples with Ti screws	Samples with Ti removal	*P**
BV/TV	0.354 ± 0.026	0.337 ± 0.062	0.496
BMD	1.882 ± 0.027	1.881 ± 0.009	0.842

Note: *Independent-sample *t*-tests were performed to compare differences between samples with and without Ti screws.
